# Challenges and Solutions during the COVID-19 Pandemic: Hospitalization and Performance in Elective Pediatric Surgeries

**DOI:** 10.3390/medicina60071072

**Published:** 2024-06-29

**Authors:** Miro Jukić, Petra Tokić, Sara Elezović Baloević, Zenon Pogorelić

**Affiliations:** 1 Department of Surgery, School of Medicine, University of Split, 21 000 Split, Croatia; 2 Department of Pediatric Surgery, University Hospital of Split, 21 000 Split, Croatia

**Keywords:** pediatric surgery, COVID-19, COVID-19 pandemic, elective surgery, healthcare, children

## Abstract

*Background and Objectives*: This retrospective study aimed to investigate the impact of the COVID-19 pandemic on the most frequently performed elective pediatric surgical procedures and the number of hospitalizations and compare it to the pre-pandemic and post-pandemic periods. *Materials and Methods*: The subjects were patients under 18 years of age who were regularly admitted for selected elective procedures in a single tertiary center in Croatia from 1 March to 31 August of 2019, 2020, 2021, and 2022. Data were collected from the electronic logs of surgical procedures and hospital admissions, logs of one-day surgeries, patients’ medical records, and discharge letters. The primary outcome of this study was to determine the evolution of the number of elective surgical procedures before, during, and after the peak of the COVID-19 pandemic; also, we aimed to confirm and objectify global data and statements about the decrease in the number of hospital admissions and surgical procedures. Secondary outcome measures included the length of hospitalization, the number and types of complications, and readmissions. *Results*: In 2020, the number of elective procedures decreased by 28.3% and the number of hospitalizations decreased by 36.2%; in 2021, the number of elective procedures decreased by 30.8% and the number of hospitalizations decreased by 14.2% compared to the pre-pandemic period (2019). In 2022, there was a 22.8% increase in elective procedures and a 2.9% decrease in hospitalizations compared to 2019. No statistical difference was found in the rates of complications between the individual years studied in terms of complications (*p* = 0.869). *Conclusions*: The number of elective procedures and hospitalizations during the COVID-19 pandemic has decreased significantly compared to the pre-pandemic period. After the healthcare system adapted to the conditions of the pandemic, the number of elective procedures increased significantly despite COVID-19, while the number of hospitalizations remained approximately the same as before the pandemic.

## 1. Introduction

On 30 January 2020, the World Health Organization (WHO) declared a global emergency due to the outbreak of the new coronavirus in a city market in Wuhan, a city in the Chinese province of Hubei [1]. The number of reported cases and deaths increased exponentially after the WHO declared the COVID-19 pandemic on 11 March 2020; on the same day, the Ministry of Health of the Republic of Croatia declared an epidemic in Croatia [2,3].

The COVID-19 pandemic posed the greatest threat to human lives, livelihoods, and the global economy since the Second World War. In addition, COVID-19 pandemic has had a significant impact on healthcare systems around the world [4,5,6,7]. Adapting healthcare systems to the epidemic led to changes in the provision of routine services, especially at the beginning of the pandemic during the exponential spread of the virus. In addition, there was the fear of infection and the possibility of transmission through family members and friends, but also the fear of a lack of COVID-19 protective clothing for healthcare staff and equipment to treat patients [6]. The pandemic has harmed both preventive and curative treatment. In primary healthcare, there has been a significant decrease in face-to-face patient visits, especially between March and June 2020, and at the same time there has been an increase in virtual consultations by e-mail or telephone [8,9]. Some studies show that the decline in doctor visits and the lower uptake of diagnostic procedures and planned therapies is partly due to patients’ fear of contracting the virus [10,11]. Hospitals worldwide have reduced elective surgical procedures and diagnostic tests in the interest of public health and to prevent hospital-acquired infections with this virus. This maintained the supply of personal protective equipment, which was of primary importance for the care of COVID-19 patients, and freed up beds for COVID-19 patients in various wards and intensive-care units [12]. As part of this reorganization, 15% of hospital beds in Croatia were adapted for COVID-19 patients [2]. Patient visits to hospital wards decreased by 60%, especially in surgical wards [2,6].

Many studies show that the number of injuries and fractures decreased in all age groups, which is primarily due to social isolation and the “stay at home” policy [13,14]. On the other hand, there were also delays in hospitalization for life-threatening illnesses or emergencies such as acute myocardial infarction, diabetic ketoacidosis, subarachnoid hemorrhage, acute appendicitis, or even testicular torsion [15,16,17,18]. Due to chronic staff shortages, surgeons and especially anesthesiologists are deployed in critical areas of hospitals to support patient care. In response to social distancing measures and travel restrictions, sessions in most surgical departments have been restricted and reduced. These include morning visits and multidisciplinary team meetings, sometimes replaced by virtual meetings as recommended [19,20,21]. With the outbreak of the COVID-19 pandemic and its rapid global spread, the Croatian healthcare system also faced enormous challenges. The results of a retrospective study showed a 21% decrease in the total number of admissions to Croatian hospitals in 2020, with a peak of 51% in April 2020 compared to the previous three years [22]. During the COVID-19 pandemic, millions of planned surgeries were canceled worldwide [23]. An estimated 28 million adult surgical procedures, equating to 72.3%, were canceled in the first 12 weeks of the pandemic. Most surgeries were canceled in Europe and Central Asia [23,24].

On the occasion of the complete lockdown in the Republic of Croatia, the government decided to suspend all elective surgical procedures, and outpatient clinics ceased to operate, except for emergencies [2,22]. If an infection was detected, elective surgery should have been postponed for at least 7 weeks [25]. Cancelation of elective surgery on this scale had a significant impact on patients and a cumulative, potentially devastating impact on healthcare systems around the world. Delaying time-critical elective surgery, such as surgery for oncology patients or transplants, can lead to deterioration in health and quality of life as well as unnecessary deaths [26]. They are time-critical in terms of the need for interventions to control pain, halt disease progression, prevent complications, and improve quality of life. In general, surgical interventions should not be delayed if a delay could harm patients, prolong their hospital stay, or predispose them to readmission [26]. The pediatric population has not been seriously affected by this virus, but due to children’s tendency to catch colds, which are difficult to distinguish from the symptoms of COVID-19, and the overall public health situation, elective procedures are being canceled. The extent to which elective procedures were affected in the pediatric population remains unclear, but an international study showed similar declines in the adult population [27]. In the initial phase of the pandemic, the guidelines of the European and North American pediatric urological societies, for example, recommended that surgical procedures during quarantine should only be performed for organ- or life-threatening conditions and recommended that all outpatient consultations should be reduced during the first wave of the pandemic [28]. A study conducted in Sweden shows a 53.7% decrease in elective procedures in the pediatric population, with the largest decrease of 72.8% in April 2020 in district hospitals, while the number of emergency surgical procedures did not change and returned to close to normal capacity by the end of June 2020 [29]. The same study found that the largest decrease was in smaller hospitals; meanwhile, in university hospitals, the decrease in elective procedures was 39.1%. This was explained by the fact that more complex pediatric patients with more severe conditions are referred to university hospitals; therefore, elective procedures are less likely to be canceled or postponed [29]. Furthermore, the Swedish healthcare strategy seems to differ from that of other countries, especially with regard to pediatric surgery. The authors explain this by the fact that the pediatric centers have quickly adapted to strict algorithms for the preoperative examination of children and that the staff in the pediatric centers were less allocated to COVID-19 wards [29]. A recent study clearly showed that pediatric surgery in large centers experienced a dramatic decline in case numbers during the lockdown, which varied by specialty. A graduated and balanced re-entry strategy was implemented, where elective surgery was continuously resumed after rigorous preoperative examinations and testing [30].

This study aimed to investigate the number, frequency, and variation of elective procedures performed in pediatric surgery in selected time periods (before the pandemic, during the pandemic, and after the pandemic). In addition, the length of hospital stay, the number and type of complications, the number of readmissions, and the number of unplanned readmissions to the operating room were investigated.

## 2. Methods

### 2.1. Patients

Pediatric patients regularly admitted to the Department of Pediatric Surgery at University Hospital of Split for planned, mostly elective surgeries in three periods from 1 March to 31 August of the years 2019, 2020, 2021, and 2022 were retrospectively included in this study. The study included all pediatric patients (aged 0–17 years) who were regularly admitted for elective surgery for the most commonly performed procedures in our department (undescended testis, inguinal hernia, umbilical hernia, pilonidal and dermal sinus, hydrocele, varicocele, phimosis, and short foreskin frenulum) during the selected study period. The above procedures were chosen as inclusion criteria because the expected duration of hospitalization, complication rates, and readmissions are more or less the same. Exclusion criteria were the following: age older than 17 years, operations for other pathologies or outside the study period, and operations on as part of emergency operations and admissions.

### 2.2. Institutional Review Board Statement

The study complied with the ethical standards of the institutional and national research committee and the 1964 Declaration of Helsinki and its subsequent amendments or comparable ethical standards, and the Institutional Review Board of the University Hospital of Split approved the study (Approval number: 500-03/22-01/172; Date of approval: 31 October 2022).

### 2.3. Outcomes of the Study and Hypotheses

The primary outcome of the study was to determine the number, frequency, and variation of elective procedures performed in pediatric surgery in selected time periods (before the pandemic, during the pandemic, and after the pandemic). Secondary outcomes were the length of hospital stay, the number and type of complications, the number of readmissions (ReAd), and the number of unplanned readmissions to the operating room (uROR) [31,32].

### 2.4. Data Collection and Study Design

The data sources for this study were the written protocols and medical archives of the Department of Pediatric Surgery of the University Hospital of Split and the Hospital Information System (IT)—IBIS IN2. The following variables were recorded for each patient: age, sex, body weight, height, body mass index (BMI), principal diagnosis, length of hospitalization, complications, and readmission. Patients were screened for surgical complications according to the Clavien–Dindo classification [33]. All variables were included in the study protocol and compared among the years studied.

### 2.5. Statistical Analysis

Statistical analyses were performed using the Statistical Package for Social Sciences 19.0 (SPSS IBM Corp., Armonk, NY, USA) and Microsoft Excel for Windows version 16.74 (Microsoft Corporation, Redmond, WA, USA) programs. Median and interquartile range (IQR) were used to describe the distribution of the quantitative data, while absolute numbers and percentages were used to describe the categorical data. The ANOVA test was used for the comparison of continuous variables. The comparison of different categorical variables was conducted using the Chi-square test. Th Fisher’s exact test was used in cases of low frequency of individual variables. All *p*-values less than 0.05 were considered significant.

## 3. Results

A total of 837 children were included in the analysis. The median age of the children was 7 years (IQR 4, 14.5). The median height of the entire cohort was 133 cm (IQR 114, 171.5), the median weight was 26 kg (IQR 17.5, 56), and the median BMI was 17.0 (IQR 15.1, 20.6). The median duration of hospital stay was 32 h (IQR 24, 44). Of the total number of patients included, 87.1% were boys, while the remaining (12.9%) were girls (*p* = 0.032). The median length of hospital stay for the selected elective procedures was 48 h in 2019 (IQR 24, 48), 24 h in 2020 (IQR 24, 48), 24 h in 2021 (IQR 24, 48), and 40 h in 2022 (IQR 24, 48), indicating a statistically significant difference between the four periods analyzed (*p* = 0.002). The complication rate was very low in the years analyzed (three complications in 2019, two in 2020, three in 2021, and six in 2022; *p* = 0.493). In 2019, one case of uROR was recorded after pilonidal sinus surgery (exploration and hemostasis), while no cases of uROR were recorded in the other years studied (*p* > 0.999). In 2022, there was one ReAd within 30 days of discharge, namely edema and bleeding after circumcision, while no cases of ReAd were recorded in the other years studied (*p* > 0.999). Demographic and clinical data of the patients are presented in Table 1.

**Figure 1:**
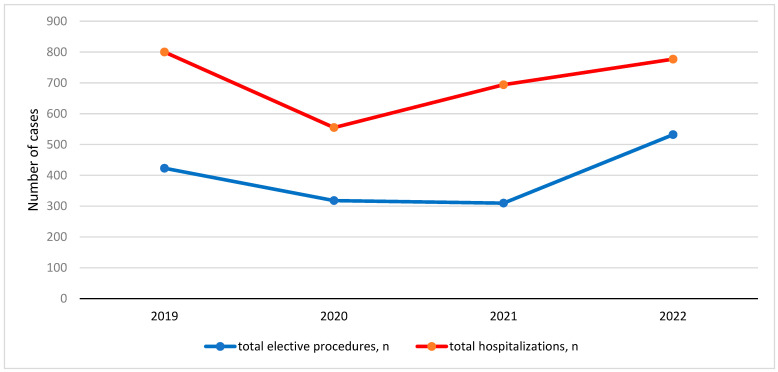
Graphical representation of the trend and oscillations in the number of total elective procedures and total hospitalizations throughout the studied period.

**Figure 2:**
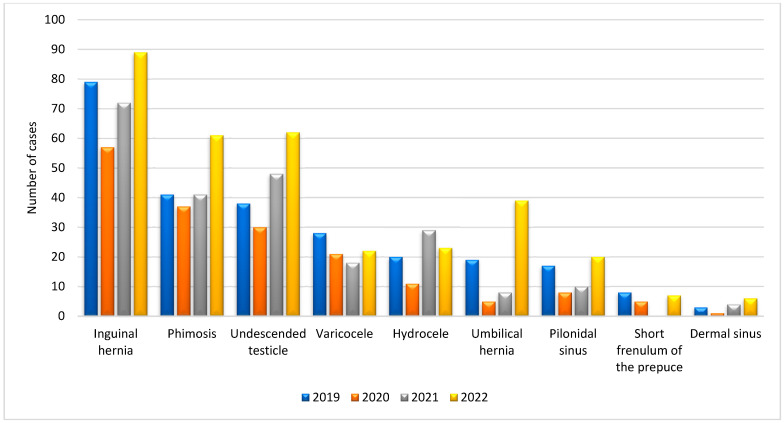
Presentation of selected elective diagnoses throughout the studied period.

According to the data presented in Table 2, the number of elective procedures decreased by 28.3% and the number of hospitalizations by 36.2% in 2020 compared to 2019 (*p* = 0.01). In 2021, the number of elective procedures decreased by 30.8% compared to 2019 and the number of hospital stays decreased by 14.2% (*p* = 0.01). In 2022, compared to 2019, there was a 22.8% increase in the number of elective procedures, a 26.11% increase in the number of selected elective procedures, and a 2.9% decrease in the number of hospitalizations (*p* = 0.01) (Figure 1 and Figure 2).

To test whether the significant variations during 2019–2022 occurred due to the COVID-19 pandemic or whether they were the result of annual fluctuations, the 2015–2018 control group was introduced. The main results examined between the groups were compared (Table 3). The number of selected elective procedures decreased by 25.5% in the second period. A similar trend was observed in the number of total elective procedures (decrease of 24.5%) and the total number of hospital stays (decrease of 28.6%). In addition, the length of stay was significantly shorter in the second period (*p* = 0.001). Although significant problems were observed in the healthcare system during the pandemic, the quality of care in our institution was good, as no differences were observed in terms of the number of complications, ReAd, or uROR between the two periods studied.

Table 4 shows the complications recorded during the study period using the Clavien–Dindo (CD) classification. In 2019, complication CD IIIa was bleeding after pilonidal sinus surgery, followed by wound exploration and hemostasis by electrocoagulation. The other two complications (CD I) of the pilonidal sinus were a seroma and a wound infection, which were treated conservatively with a wound dressing, wound cleansing, and antibiotic therapy. In 2020, two complications (CD I) were a seroma following pilonidal sinus surgery and a post-circumcision hemorrhage that was treated conservatively with a wound dressing. In 2021, two complications (CD I) were a wound infection following surgical treatment of a pilonidal sinus and a recurrence following circumcision. Complication IIIa (CD) was a recurrence of a pilonidal sinus that was operated on again. In 2022, the three complications CD I were edema and swelling after varicocelectomy. The other three complications IIIa were one recurrence after partial circumcision and two recurrences of pilonidal sinus, and these patients underwent re-operation. No statistical difference in rates of complications was found between the individual years studied in terms of complications (*p* = 0.869).

## 4. Discussion

The aim of this study was to present the impact of the COVID-19 pandemic on the performance of elective surgical procedures and hospitalizations in the Pediatric Surgery Clinic of our hospital during the four periods studied: firstly, before the pandemic (2019); secondly and thirdly, during the period when changes were initiated and the lockdown occurred (2020 and 2021); fourthly, during the last period when clinical capacities were returned to the standard (2022). This study also examined the impact on length of hospital stay, the number of complications, and the number of re-hospitalizations. To confirm the first study hypothesis, our results show that 245 more patients were hospitalized in 2019 than in 2020; 106 more patients were hospitalized in 2019 than in 2021. In addition, 23 more patients were hospitalized in 2022 than in 2019, which confirms our second study hypothesis, namely that the number of hospitalizations has reached or/and is returning to pre-pandemic rates.

As our first study hypothesis shows, elective surgeries have decreased sharply in number and rate: 28.3% less in 2020 and 30.8% less in 2021 than in 2019. In 2022, there was a 22.8% increase in elective surgical procedures compared to 2019, confirming our second study hypothesis. Our results are consistent with other studies worldwide, for example, in Sweden during the first wave, the number of pediatric elective cases decreased by 53.7% and surgical capacity recovered to near-normal levels by the end of June 2020 [29]. In addition, the results of a study on the impact of COVID-19 on the number of procedures at a tertiary children’s hospital in Seattle showed a 55% decrease in all procedures requiring anesthesia in 2020 compared to 2019 [34]. Another study recorded a 32% decrease in total surgical procedures from March to April 2020 in four pediatric hospitals worldwide [35]. The same study shows that the patients hospitalized during this period were younger; meanwhile, in our study, no statistically significant difference was recorded [35]. The available studies in Croatia show a 21% decrease in the total number of hospital admissions across Croatia in 2020, peaking at 51% in April 2020 [22]. In the Department of Otorhinolaryngology and Head and Neck Surgery at the University Hospital of Zagreb, a total decrease in surgical procedures of 27% was recorded in 2020 [36]. In 2019, 79 surgical interventions for hernias were recorded in the Pediatric Surgery Clinic of the University Hospital Split in the period studied, and 57 were recorded in 2020. These collected surgical data are consistent with a retrospective study conducted at the Royal Manchester Children’s Hospital, where 76 surgical interventions for hernia repair were recorded in a similar period (from 1 April to 30 September), 46 of which were performed in 2019 in the same period [37].

The COVID-19 pandemic has had a significant impact on healthcare systems around the world, dramatically changing the availability of medical care for patients and the quality of life of patients. A balance should be struck between measures to combat epidemics and ensuring adequate care for patients. This has been particularly evident in limiting the number of elective surgical procedures and shortening hospital stays. A multicenter, retrospective cohort study analyzed data from patients undergoing general surgical procedures, such as ventral hernia repair, in hospitals participating in the American College of Surgeons; they showed a 21% increase in same-day discharge rates compared to patients who underwent the same procedures the year before [38]. The authors also emphasized an increase in same-day discharge for other common surgical procedures as well and the accelerated transition from inpatient to outpatient general surgery [38]. These data can be compared with our data, which show that the median length of stay in hospital was 24 h in 2020 and 2021, while it was 48 h in 2019 and 40 h in 2022. In addition, the COVID-19 pandemic has also drastically changed and shortened the median length of hospital stay for some acute illnesses. Until 2019, the one-day surgery system did not formally exist at our facility, but at our insistence, it was contracted with the appropriate insurance company. Therefore, in the initial years, a lower number of patients underwent operations on the basis of one-day surgery, as we needed the time to provide staff and redistribute accommodation capacities. Recently, the vast majority of children with the above-mentioned diagnoses are treated exclusively in one-day surgery. Another, more important reason is that our hospital covers a wide area that includes a large number of islands and remote locations; for their populations, one-day surgeries are not suitable due to distance and transportation problems. Children of these populations are either admitted to hospital on the day of surgery or earlier; this is because transportation is not possible the day before surgery, which increases the average length of hospital stay. A recent study conducted at our institution has clearly shown that even patients with non-complicated appendicitis can be safely discharged within 24 h. In this study, the median length of hospital stay was 15 h (IQR 12 to 19 h), and only 2.2% of patients had an unplanned ReAd before the seventh postoperative day due to postoperative complications; these data are very similar to those of patients who stayed in the hospital for two–three days after surgery. The level of parental satisfaction with this protocol was very high [39]. Furthermore, Habbous et al. demonstrated in their study that patients who underwent outpatient arthroplasty had relatively similar outcomes to patients who underwent inpatient surgery after matching with known sociodemographic and clinical characteristics [40]. Siron et al. also confirmed that same-day discharge for robotic radical prostatectomy or percutaneous nephrolithotomy is safe and feasible in selected patients, with an acceptable complication rate [41].

Recent studies dealing with pediatric surgical procedures have shown similar results and indicate that pediatric surgery should return to normality as soon as possible; longer waiting lists for operations have formed and potential problems arise from delayed operations. The number of outpatient examinations was reduced, especially for the first examinations, and then some patients’ cases were so progressed that they had to undergo surgery instead of conservative treatment [42,43,44].

In addition, our study demonstrates significant difference in age groups. Children operated on during the lockdown were significantly younger compared to the children of other years analyzed. 

We assume that this age difference is only coincidental due to the significant decrease in elective surgeries during the lockdown. During lockdown, we attempted to operate on children with undescended testes or recurrent hernia incarceration to avoid testicular atrophy or bowel necrosis. Other surgeries included in this study that are more common in adolescents, such as varicocelectomy or pilonidal sinus surgery, were not the highest priority. No statistical significance was achieved with regard to differences in weight, height, or BMI, which is probably due to the sample size. With a larger sample, this difference would likely be greater.

The main limitations of this study were the single-center design and the retrospective approach to data collection, which may have led to deficiencies in the demographic and clinical data of the selected patients. Therefore, the data collected cannot be considered to be internationally representative, and may not be generalizable to other centers worldwide as they exclude pediatric cardiac and neurosurgical operations. In addition, differences in the provision of epidemiologic measures and surgical protocols worldwide should be considered. Further studies and meta-analyses should be conducted to substantiate these data.

## 5. Conclusions

This retrospective study confirms a significant decrease in the number of elective surgical procedures and the total number of hospitalizations at our center during the COVID-19 pandemic, which is consistent with other studies at surgical centers worldwide. On the other hand, our results showed a gradual recovery in the number of elective surgical procedures and hospitalizations to pre-pandemic levels.

## Figures and Tables

**Table 1:** Demographic and clinical data of the patients.

Year/Variables		2019	2020	2021	2022	*p*
Sex	Male; *n* (%)	182 (87.5)	135 (93.1)	175 (88)	237 (83.2)	0.032 *
	Female; *n* (%)	26 (12.5)	10 (6.9)	24 (12)	48 (16.8)	
Age (years); median (IQR)		8 (4, 15)	8 (4, 15)	6 (4, 11)	7 (5, 15)	0.013 ^†^
Height (cm); median (IQR)		130 (113, 173.5)	135 (106, 173)	127 (112, 150)	134 (110, 174)	0.101 ^†^
Weight (kg); median (IQR)		26 (17, 59.5)	27 (16.5, 55)	25 (17, 40)	26 (18, 60)	0.063 ^†^
BMI (kg/m^2^); median (IQR)		17.1 (15.2, 20.5)	17.9 (16, 21)	16.7 (14.9, 19.5)	17.6 (14.8, 21.1)	0.071 ^†^
Complications; *n* (%)		3 (1.2)	2 (1.1)	3 (1.3)	6 (1.8)	0.493 ^‡^
LOS; median (IQR)		48 (24, 48)	24 (24, 48)	24 (24, 48)	40 (24, 48)	0.002 ^†^
ReAd; *n* (%)		0	0	0	1 (0.35)	>0.999 ^‡^
uROR; *n* (%)		1 (0.5)	0	0	0	>0.999 ^‡^

IQR—interquartile range; BMI—body mass index; LOS—length of hospital stay; ReAd—readmission to hospital within 30 days; uROR—unplanned return to operating room. * Chi-square test; ^†^ ANOVA test; ^‡^ Fisher’s exact test.

**Table 2:** Presentation of the number of the most common elective diagnoses, hospital stays, and the total number of elective procedures during the study period.

Diagnosis/Year; *n* (%)	2019	2020	2021	2022
Inguinal hernia	79 (31.2)	57 (32.6)	72 (31.3)	89 (27)
Phimosis	41 (16.2)	37 (21.1)	41 (17.8)	61 (18.5)
Undescended testicles	38 (15)	30 (17.1)	48 (20.9)	62 (18.8)
Varicocele	28 (11.1)	21 (12)	18 (7.8)	22 (6.7)
Hydrocele testis	20 (7.9)	11 (6.3)	29 (12.6)	23 (7)
Umbilical hernia	19 (7.5)	5 (2.9)	8 (3.5)	39 (11.9)
Pilonidal sinus	17 (6.7)	8 (4.6)	10 (4.4)	20 (6.1)
Short foreskin frenulum	8 (3.2)	5 (2.9)	0 (0)	7 (2.1)
Dermal sinus	3 (1.2)	1 (0.5)	4 (1.7)	6 (1.8)
**Total; *n***	**2019**	**2020**	**2021**	**2022**
Selected elective procedure	253	175	230	329
Hospitalized patients due to selected elective procedures	208	145	199	285
Total elective procedures	423	318	310	532
Total hospitalizations	800	555	694	777

**Table 3:** Comparison of demographic data and investigated outcomes of treatment between the study group (2019–2022) and control group (2015–2018).

Period/Variables		2015–2018 (*n* = 1124)	2019–2022 (*n* = 837)	*p*
Gender	Male; *n* (%)	994 (88.4)	729 (87)	0.369 *
	Female; *n* (%)	130 (11.6)	108 (13)	
Age (years); median (IQR)		8 (5, 15)	7 (4, 14.5)	0.064 ^†^
Height (cm); median (IQR)		137 (119, 177.5)	133 (114, 171.5)	0.095 ^†^
Weight (kg); median (IQR)		28.5 (20, 61)	26 (17.5, 56)	0.204 ^†^
BMI (kg/m^2^); median (IQR)		17.8 (16.2, 21.4)	17.0 (15.1, 20.6)	0.151 ^†^
Complications; *n* (%)		17 (1.5)	14 (1.7)	0.778 *
LOS; median (IQR)		48 (24, 48)	32 (24, 44)	0.001 ^†^
ReAd; *n* (%)		3 (0.3)	1 (0.1)	0.640 ^‡^
uROR; *n* (%)		2 (0.2)	1 (0.1)	>0.999 ^‡^
**Period/Variables**		**2015–2018**	**2019–2022**	**↙ %**
Selected elective procedure; *n*		1124	837	↙ 25.5%
Total elective procedures; *n*		2097	1583	↙ 24.5%
Total hospitalizations; *n*		3958	2826	↙ 28.6%

IQR—interquartile range; BMI—body mass index; LOS—length of hospital stay; ReAd—readmission to hospital within 30 days; uROR—unplanned return to operating room. * Chi-square test; ^†^ Mann–Whitney-U test; ^‡^ Fisher’s exact test.

**Table 4:** Presentation of complications according to the Clavien–Dindo (CD) classification.

Clavien–Dindo Grade	2019	2020	2021	2022	*p*
I	2	2	2	3	0.896 *
II	0	0	0	0	
IIIa	1	0	1	3	
IIIb	0	0	0	0	
IVa	0	0	0	0	
IVb	0	0	0	0	
V	0	0	0	0	

* Fisher’s exact test.

## Data Availability

The data assessed and reported here can be obtained from the authors upon reasonable request and following ethical and privacy principles.
